# Loss of sphingosine kinase 1 increases lung metastases in the MMTV-PyMT mouse model of breast cancer

**DOI:** 10.1371/journal.pone.0252311

**Published:** 2021-05-27

**Authors:** Fabiola N. Velazquez, Leiqing Zhang, Valentina Viscardi, Carolena Trocchia, Yusuf A. Hannun, Lina M. Obeid, Ashley J. Snider

**Affiliations:** 1 Department of Medicine, Stony Brook University, Stony Brook, NY, United States of America; 2 Cancer Center, Stony Brook University, Stony Brook, NY, United States of America; 3 Department of Nutritional Sciences, College of Agriculture and Life Sciences, University of Arizona, Tucson, AZ, United States of America; University of Tennessee Health Science Center, UNITED STATES

## Abstract

Breast cancer is a very heterogeneous disease, and ~30% of breast cancer patients succumb to metastasis, highlighting the need to understand the mechanisms of breast cancer progression in order to identify new molecular targets for treatment. Sphingosine kinase 1 (SK1) has been shown to be upregulated in patients with breast cancer, and several studies have suggested its involvement in breast cancer progression and/or metastasis, mostly based on cell studies. In this work we evaluated the role of SK1 in breast cancer development and metastasis using a transgenic breast cancer model, mouse mammary tumor virus-polyoma middle tumor-antigen (MMTV-PyMT), that closely resembles the characteristics and evolution of human breast cancer. The results show that SK1 deficiency does not alter tumor latency or growth, but significantly increases the number of metastatic lung nodules and the average metastasis size in the lung of MMTV-PyMT mice. Additionally, analysis of Kaplan-Meier plotter of human disease shows that high SK1 mRNA expression can be associated with a better prognosis for breast cancer patients. These results suggest a metastasis-suppressing function for SK1 in the MMTV-PyMT model of breast cancer, and that its role in regulating human breast cancer progression and metastasis may be dependent on the breast cancer type.

## Introduction

Of all types of cancer, breast cancer is the most common type among women. In 2018, breast cancer had an incidence of 11.6% among all types of cancer, accounting for 6.5% of mortalities worldwide [1]. In the United States, breast cancer incidence rate has increased by 0.3% per year over the most recent 5-year period (2012–2016), but the rate of death has dropped 40% from 1989 to 2017 [2]; a decline that can be explained by advancements in early screening and therapy [3]. Breast cancer treatment is based on the receptor status of the tumor, specifically estrogen receptor (ER), progesterone receptor (PR) and human epidermal growth factor receptor-2 (HER2), as well as proliferative markers such as Ki67. The main breast cancer molecular subtypes are termed Luminal A (ER+/PR+/HER2-/low Ki-67); Luminal B (ER+/PR+/HER2-/+/high Ki-67); HER2-overexpression (ER-/PR-/HER2+) and basal/triple negative breast cancers (TNBCs) (ER-/PR-/HER2-) [4].

Multiple treatment options are available for patients with ER+ and HER2+ tumors; however, the TNBC subgroup, which accounts for ~15% of all breast cancers, lacks a specific therapeutic target [5, 6]. TNBC patients receive chemotherapy but have an increased risk of relapse and distant metastasis in the first 3–5 years following diagnosis. Thirty percent of breast cancer patients do not survive the metastatic disease; therefore, the development of anti-metastatic therapies is crucial to overall patient survival [7]. Intensive research efforts are necessary to develop novel breast cancer therapeutics with minimal negative impact on health-related quality of life. Therefore, it is imperative to examine the mechanisms involved in breast cancer progression in order to identify novel therapeutic targets.

In recent years, sphingolipids have emerged as key signaling molecules that regulate normal physiology as well as processes involved in cancer development and progression [8]. Sphingolipid metabolism is complex and generates several unique bioactive molecules through the action of different enzymes. Ceramide, sphingosine, and sphingosine 1 phosphate (S1P) are three of the most studied sphingolipids involved in many biological processes within the cell, including regulation of survival, proliferation, differentiation, and apoptosis. Ceramide is deacylated to generate the pro-apoptotic lipid sphingosine by ceramidases, and sphingosine can be phosphorylated to form the anti-apoptotic lipid S1P by the action of sphingosine kinases (SKs), of which there are two isoforms, SK1 and SK2. The balance between levels of ceramide, sphingosine and S1P is thought to be important in determining cell fate decisions (survival or cell death) [9]. In particular, SK1 has been shown to be a critical enzyme regulating the levels of these sphingolipids [10], thereby making it an essential enzyme controlling cell fate through a variety of characterized signaling pathways.

SK1 has been shown to be upregulated in patients with breast cancer, and its expression correlates with cancer progression and poor prognosis [11, 12]. Additionally, high SK1 mRNA and protein levels are associated with metastasis and poor response to chemotherapy [13–15]. In ERα-positive human MCF7 breast cancer cells, SK1 has been shown to confer a growth advantage by an estrogen-dependent mechanism related to activation of extracellular signal-related kinases (ERK1/2) [16]. Furthermore, SK1 overexpression promotes tumorigenesis of MCF7 cells in nude mice and increases neovascularization of the tumors [16]. Proliferation and migration of MCF7 cells after prolactin or estradiol treatment was shown to be dependent on SK1 activation [17, 18]. Also, SK1 activation was determined essential in EGF-induced MCF7 cell migration and proliferation [19]. In TNBC cells, SK1 has been shown to participate in the promotion of cell migration, invasion and metastasis in cell culture and xenograft models [20, 21]. Recent work has reported that SK1 drives TNBC cell invasion by upregulating gene expression of FSCN1 (fascin 1) which is a protein involved in the assembly of actin filament bundles [22]. In the same work, high SK1 and FCSN1 expression in TNBC tissues correlated with an increase in distant metastasis and poor survival in TNBC patients [21]. Only one study has reported on the role of SK1 *in vivo* models of breast cancer where it was shown that SK1 deficiency decreases breast tumor growth in MMTV-neu transgenic induced breast cancer, but analysis of metastasis was not documented in that study [23]. Although additional *in vivo* studies, using xenografts and syngeneic mouse models, support the involvement of SK1 in breast cancer progression and metastasis [21, 24], there are no other reports assessing the role of SK1 in genetically engineered mouse models with spontaneous breast tumor initiation within the correct microenvironment from an otherwise normal mammary cell, and with an intact immune system.

In the present work we evaluated the role of SK1 in breast cancer development and metastasis using a transgenic breast cancer model, mouse mammary tumor virus-polyoma middle tumor-antigen (MMTV-PyMT). MMTV-PyMT mice exhibit a short latency of tumor formation and a high frequency of pulmonary metastasis. The tumors formed in this model closely resemble the characteristics of human breast cancer [25], progressing through all the stages from adenoma formation to high-grade cancer with high metastatic potential [26]. Even though SK1 deficient (SK1^-/-^) [27] and MMTV-PyMT [28] mice have been available for more than 15 years, the generation of MMTV-PyMT SK1^-/-^ mice to evaluate the effects of SK1 absence in spontaneous breast cancer development and metastasis has not yet been described. Here we report on the unexpected finding that SK1 knock out mice exhibit increased lung metastasis in this model.

## Materials and methods

### Mouse models

MMTV-PyMT (FVB/N-Tg (MMTV-PyMT)634Mul/J) mice were purchased from The Jackson Laboratory. SK1^-/-^ mice were previously described [27]. SK1^-/-^ mice were backcrossed for ten generations with FVB mice, and then crossed with MMTV-PyMT mice to obtain MMTV-PyMT SK1^+/+^ and MMTV-PyMT SK1^-/-^ female mice for this study. No obvious health problems were observed in SK1^-/-^ /FVB background mice. Animals were maintained under standard laboratory conditions and monitored daily for overall health. Mice were monitored twice weekly for normal behavior and were removed from the study if they exhibited impaired ambulation, difficult or labored breathing, ulcerated tumors or the inability to remain upright. Approximately 3% of mice were removed from the study due to these humane endpoints. The number of mice used for the study were based on data in the literature, and our previous experience with this model [29]. All animal procedures were approved by the Institutional Animal Care and Use Committee at and Stony Brook University (SBU) and followed the guidelines of the American Veterinary Medical Association.

### Tumor monitoring

From 8 weeks of age, female mice were observed visually twice per week to monitor the onset of mammary tumors. Tumor size was monitored and measured every two weeks using digital calipers. Mice were euthanized at 16 weeks of age and whole blood collected via cardiac puncture. Tumors were excised and weighed, and tumor volume was calculated using (length x width^2) x 0.52. Tumors were fixed in 10% buffered formalin or snap frozen in liquid nitrogen for further studies. Lungs from 16 week old mice were inflated with 1.5 ml of 10% buffered formalin via tracheal injection. Once inflated, lungs were checked for surface metastatic foci. Fixed lungs were serially sectioned 5 mm apart and stained with hematoxylin and eosin. The number of lung metastatic foci was counted by microscopy, and their sizes were measured using NIH Image J software. The volume (mm^3^) for each lung nodule was calculated using the following formula: 1/2 × longest diameter × smallest diameter × smallest diameter.

### LC-MS/MS sphingolipid analysis

Whole blood was mixed with 2 ml RPMI and then directly lysed with 2 ml media extraction mixture (15:85 isopropanol/ethyl acetate). Samples were spiked internal standard (50pmol) containing C17-sphingosine and extracts were then analyzed by the Lipidomic Core Facility at Stony Brook University Medical Center, as described previously [30]. Lipid levels were normalized to volume.

### Complete blood counts

Whole blood was collected in EDTA tubes and complete blood counts analyzed on a (Drew Scientific Mascot HemaVet 950FS Veterinary Hematology Analyzer).

### RNA extraction and quantitative real-time PCR

RNA extraction was performed using the PureLink RNA Mini Kit following the manufacturer’s protocol. 1 μg of RNA was then used for cDNA synthesis using the qScript cDNA SuperMix according to the manufacturer’s protocol.

Real-time PCR was carried out using the Applied Biosystems 7500 Real-Time PCR System (Applied Biosystems, Foster City, CA, USA). The following TaqMan probes (Thermo Fisher Scientific) were used: mouse SK1 (ID: Mm00448841_g1) and mouse β-actin (ID: Mm00607939_s1) as a housekeeping gene. Cycle threshold (Ct) values were obtained for each gene of interest and β-actin. ΔCt values were calculated, and the relative gene expression normalized to control samples was calculated from ΔΔCt values.

### KM-Plotter analysis

The association between the SK1 mRNA expression levels of individuals with breast cancer and relapse free survival (RFS) was analyzed using KM-Plotter database [31]. Cohorts of patients were split by median expression values through auto select best cut-off. A collection of clinical data, including all breast cancer patients, luminal A and luminal B breast cancer subtypes were analyzed.

### Statistical analysis

Statistical analyses were performed using GraphPad Prism (GraphPad Software). Data are presented as mean ± SD. and were analyzed by two-tailed, unpaired Student’s t test. p<0.05 was considered statistically significant.

## Results

### SK1 deficiency does not affect primary breast tumor growth in MMTV-PyMT mice

**Fig 1:**
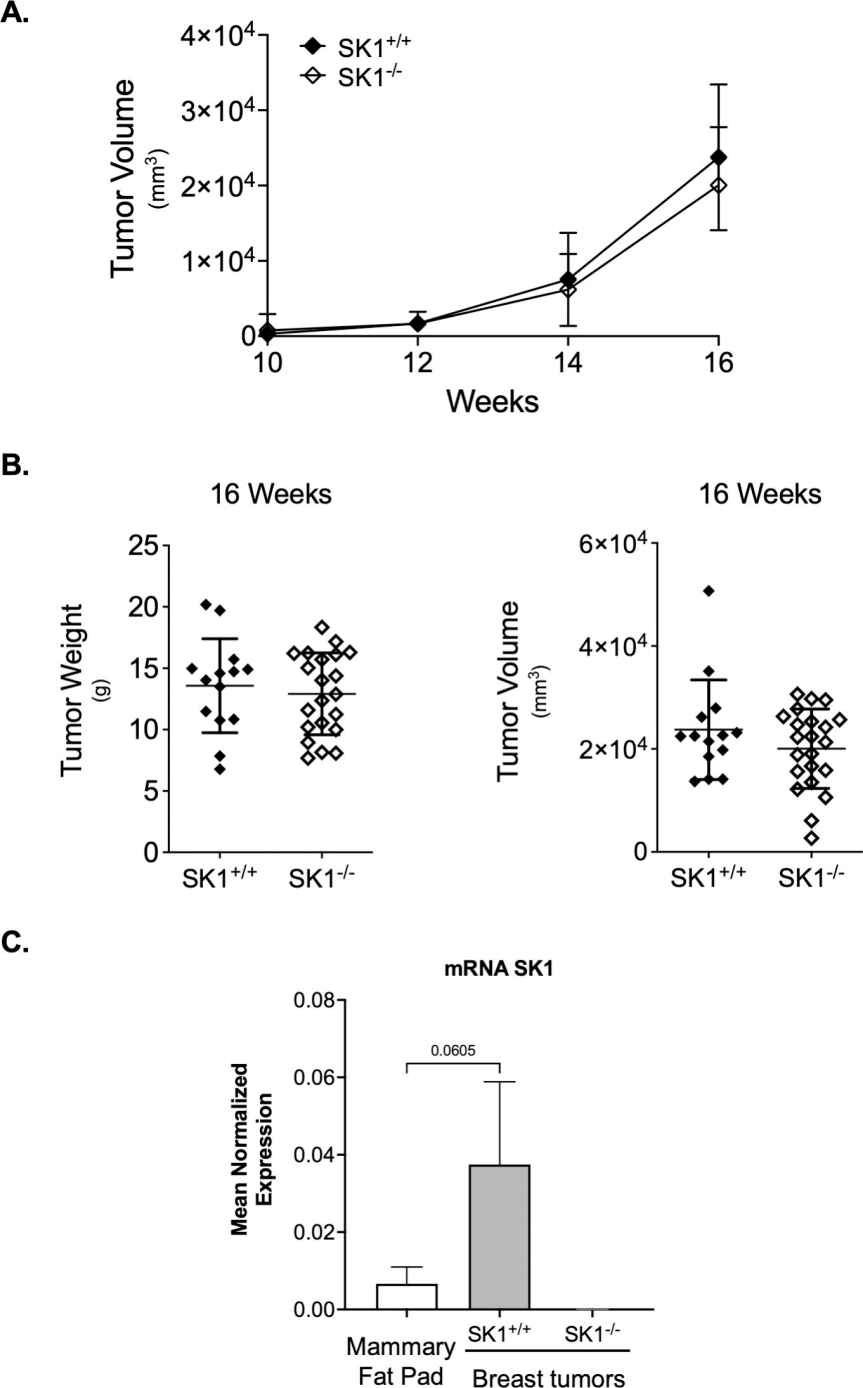
SK1 deficiency does not affect primary breast tumor growth in MMTV-PyMT mice. **A.** Tumor growth over time in age-matched transgenic mice. Tumor volume was measured in MMTV-PyMT SK1^+/+^ and MMTV-PyMT SK1^-/-^ mice at 10, 12, 14 and 16 weeks of age with calipers. Data are plotted as the mean ± SD. **B.** Tumor weight and volume in MMTV-PyMT SK1^+/+^ (n = 14) and MMTV-PyMT SK1^-/-^ (n = 21) mice after euthanasia at 16 weeks of age. Data are plotted as the mean ± SD. **C**. SK1 expression in mammary fat pad from MMTV-PyMT SK1^+/+^ mice at 8 weeks of age (n = 4) and in breast tumors from MMTV-PyMT SK1^+/+^ mice at 16 weeks of age (n = 4). Breast tumors MMTV-PyMT SK1^-/-^ mice at 16 weeks of age (n = 4) were used as negative controls. Data are plotted as mean ± SD. Actin expression was used as an internal control for normalization.

In order to define the role of SK1 in breast cancer in an *in vivo* model of spontaneous generation of breast tumors, female MMTV-PyMT SK1^-/-^ mice were generated with MMTV-PyMT SK1^+/+^ used as controls. Starting at 8 weeks of age, mice were monitored every week to detect breast tumor formation and to assess overall health. Once tumor formation was detected, tumor volume was measured every week. No differences were observed in tumor latency or tumor growth over time between MMTV-PyMT SK1^-/-^ and MMTV-PyMT SK1^+/+^ mice (Fig 1A), suggesting that SK1 is not participating in the *in vivo* breast tumor progression in MMTV-PyMT mice. Likewise, there were no differences in tumor weight or total tumor volume at week 16 (Fig 1B). SK1 expression was evaluated in mammary fat pad and breast tumors from MMTV-PyMT SK1^+/+^ mice at 8 and 16 weeks of age respectively, to confirm its expression in tumor tissue from MMTV-PyMT mice (Fig 1C). These data suggest that SK1 expression does not regulate the *in vivo* breast tumor progression in MMTV-PyMT mice.

### Absence of SK1 increases lung metastasis in MMTV-PyMT mice

**Fig 2:**
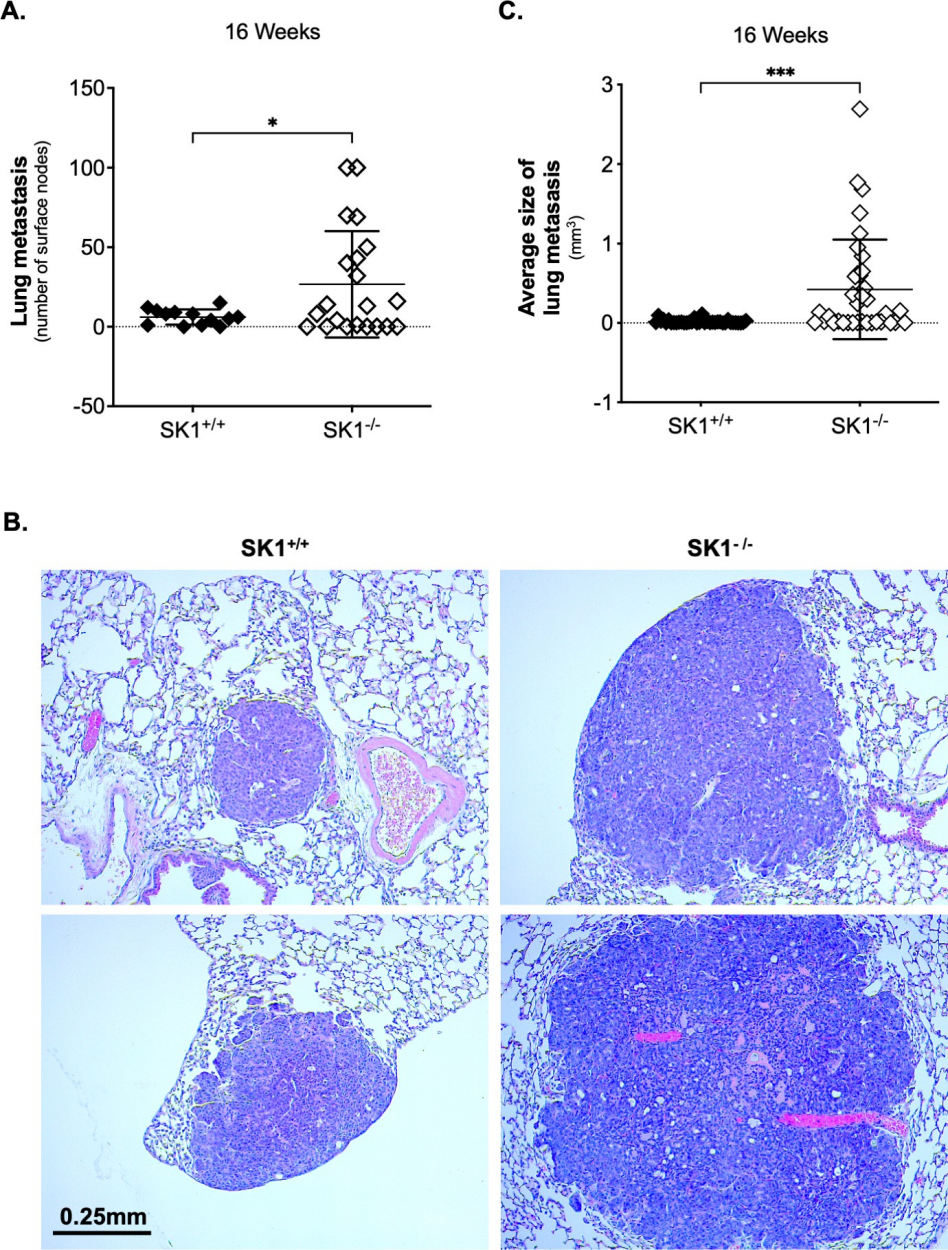
Absence of SK1 increases lung metastasis in MMTV-PyMT mice. **A.** Quantification of the number of surface nodes in lung from 16 weeks old MMTV-PyMT SK1^+/+^ and MMTV-PyMT SK1^-/-^ mice. Data are plotted as the mean ± SD. Statistical analysis: Unpaired t test, *p < 0.05, n = 13 for SK1^+/+^ and 21 for SK1^-/-^. **B.** Quantification of the average size of lung metastasis from 3 mice per genotype. Data are plotted as the mean ± SD. Statistical analysis: Unpaired t test with Welch’s correction, ***p < 0.001, n = 12 metastases per genotype were analyzed. C. Representative microscopic images of hematoxylin and eosin stained lung sections from MMTV-PyMT SK1^+/+^ and MMTV-PyMT SK1^-/-^ mice. Magnification = x10, scale bar = 0.25mm.

MMTV-PyMT mice are characterized by short latency and a high incidence of lung metastasis, and SK1 has been previously reported to play a key role in driving migration and metastasis of breast cancer cells [20, 21]. Therefore, the effect of SK1 deficiency on lung metastasis in the MMTV-PyMT breast cancer model was analyzed. After mice were euthanized at week 16, lungs were inflated and fixed with 10% buffered formalin and the number of surface nodes counted. A higher number of surface nodes were observed in the MMTV-PyMT SK1^-/-^ (26.67 ± 33.47) compared to MMTV-PyMT SK1^+/+^ (6.08 ± 4.81) mice (Fig 2A), suggesting an increase in lung metastasis in SK1 deficient mice. Additionally, the average metastasis size in MMTV-PyMT SK1^-/-^ was significantly higher compared to control mice (Fig 2B). These results indicate that although SK1 does not affect primary breast tumor growth, loss of SK1 in a genetic model increases metastasis in the MMTV-PyMT model.

### Analysis of circulating leukocytes in MMTV-PyMT SK1^-/-^ and MMTV-PyMT SK1^+/+^ mice

**Fig 3:**
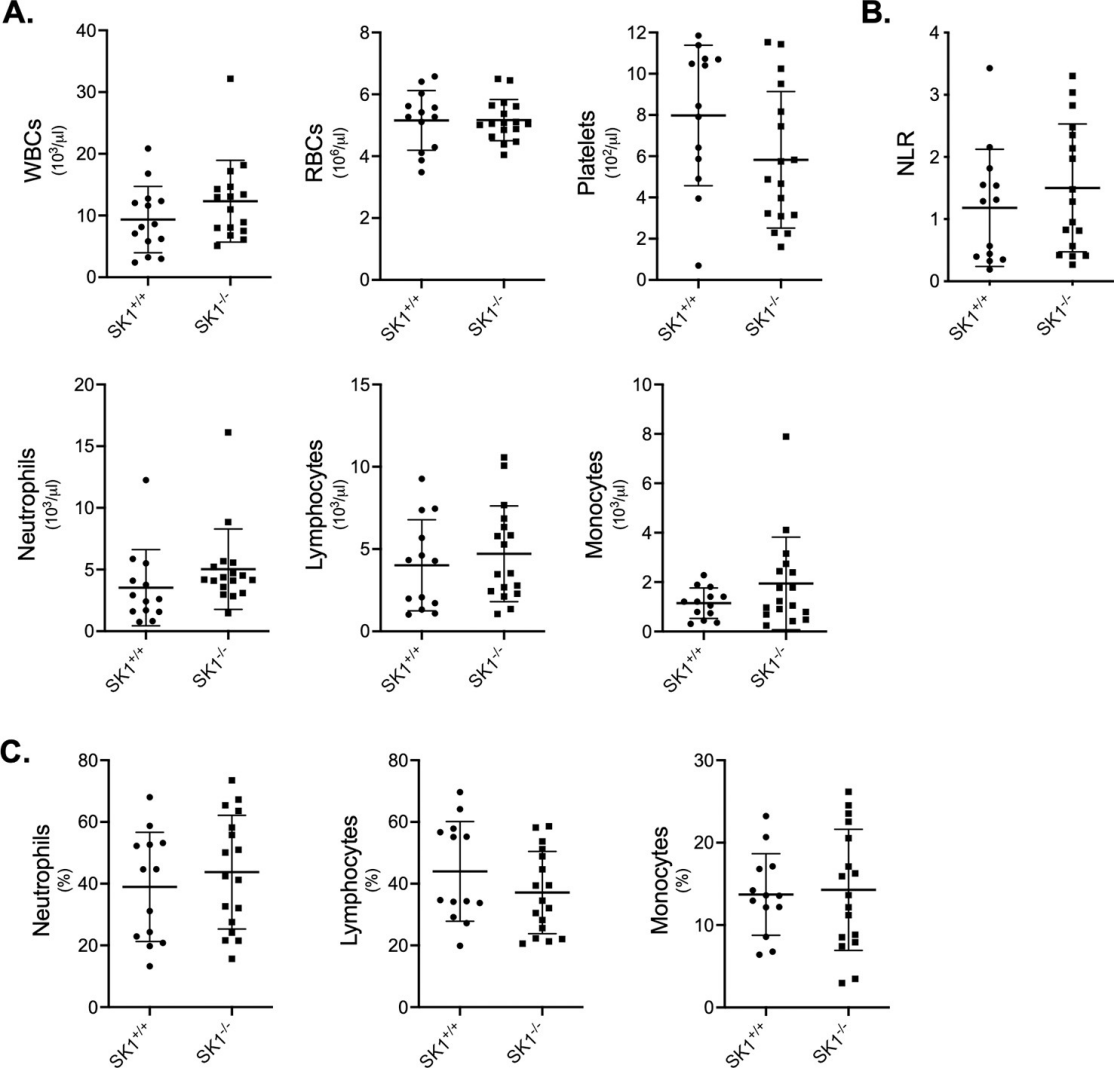
Circulating leukocytes in MMTV-PyMT SK1^-/-^ and MMTV-PyMT SK1^+/+^ mice. **A.** Blood counts were performed on whole blood from MMTV-PyMT SK1^+/+^ and MMTV-PyMT SK1^-/-^ mice at weeks 16 of age. Blood was analyzed for white blood cells (WBCs), red blood cells (RBCs), platelets, neutrophils, lymphocytes and monocytes number. Data represent mean ± SD. N = 13 for SK1^+/+^ and 17 for SK1^-/-^. **B**. The Neutrophil/Lymphocyte ratios (NLR) for MMTV-PyMT SK1^+/+^ and MMTV-PyMT SK1^-/-^ mice were calculated and represented as mean ± SD. N = 13 for SK1^+/+^ and 17 for SK1^-/-^. **C.** Percentage of neutrophils, lymphocytes and monocytes in blood. Data represent mean ± SD. N = 13 for SK1^+/+^ and 17 for SK1^-/-^.

Increased levels of leucocytes and their subtypes provide a measurable parameter that reflects the systemic inflammatory response associated with metastasis progression [32]. To compare the different cell blood populations between MMTV-PyMT SK1^-/-^ and MMTV-PyMT SK1^+/+^ mice, whole blood was collected at 16 weeks of age, and blood cells were quantified. No significant differences in white blood cells, neutrophils, lymphocytes, monocytes, platelets and red blood cells number were observed (Fig 3A). Additionally, the Neutrophil/Lymphocyte ratio, a parameter associated with breast cancer prognosis [33] was calculated for each mouse but no differences were observed (Fig 3B). Importantly, both strains showed an increase in WBCs compared to normal females [34] (normal range: 5700±800, determined in FVB/NJ female mice between the age of 12 to 16 weeks, n = 15) reflecting a systematic inflammatory response. Also, both strains showed an increased percentage of neutrophils and monocytes, and a decreased percentage of lymphocytes (Fig 3C) compared to normal ranges [34]. These results suggest that SK1 deletion does not significantly alter the inflammatory response, in terms of leukocyte number, during cancer progression in the MMTV-PyMT model.

### Blood sphingolipid levels in MMTV-PyMT SK1^-/-^ and MMTV-PyMT SK1^+/+^ mice

**Fig 4:**
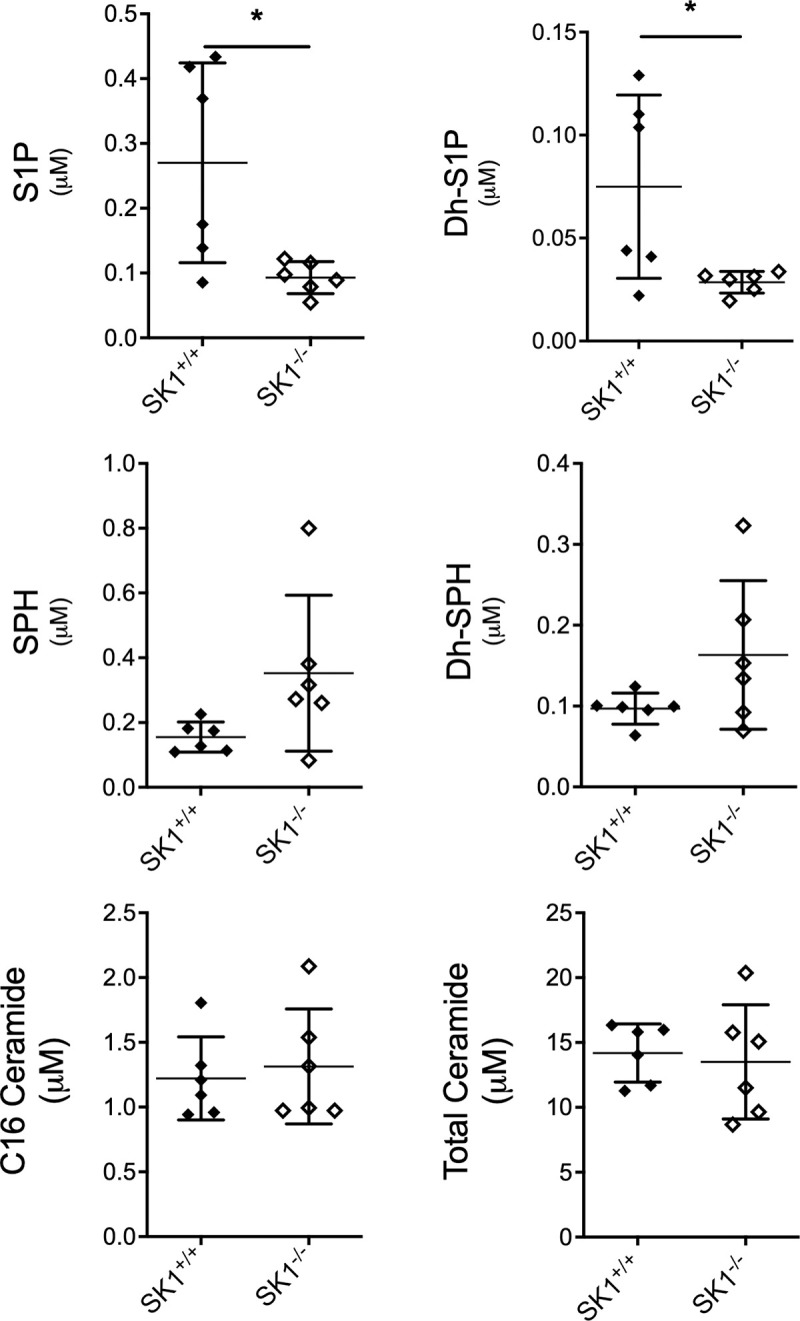
SK1 deficiency decreased S1P and DhS1P levels in blood of MMTV-PyMT mice. Whole blood was collected with anticoagulant from mice at 16 weeks of age. Samples were analyzed for sphingolipid content using LC-MS/MS by the Lipidomic Core Facility at Stony Brook University Medical Center. Data represent mean ± SEM. Statistical analysis: Unpaired t test, *p < 0.05, n = 6 for SK1^+/+^ and SK1^-/-^. SPH, sphingosine; DhSPH, dihydro-sphingosine; S1P, sphingosine 1 phosphate; DhS1P, dihydro-sphingosine 1 phosphate.

S1P has been shown to have a critical role in endothelial cell barrier function [35]. Due to the critical position of SK1 in the sphingolipid metabolic pathway and its determined role in the maintenance of blood S1P levels, sphingolipid levels were evaluated in the blood of MMTV-PyMT SK1^-/-^ and MMTV-PyMT SK1^+/+^ mice. The lack of SK1 expression resulted in a selective decrease in S1P and dhS1P levels compared to SK1^+/+^ mice (Fig 4). No significant changes in other sphingolipids species were observed. These results reflect the absence of SK1 expression in the MMTV-PyMT SK1^-/-^ mice and raise the possibility that this decrease in blood S1P levels may be involved in the increased lung metastasis observed.

### Analysis of SK1 mRNA expression in breast cancer patients

**Fig 5:**
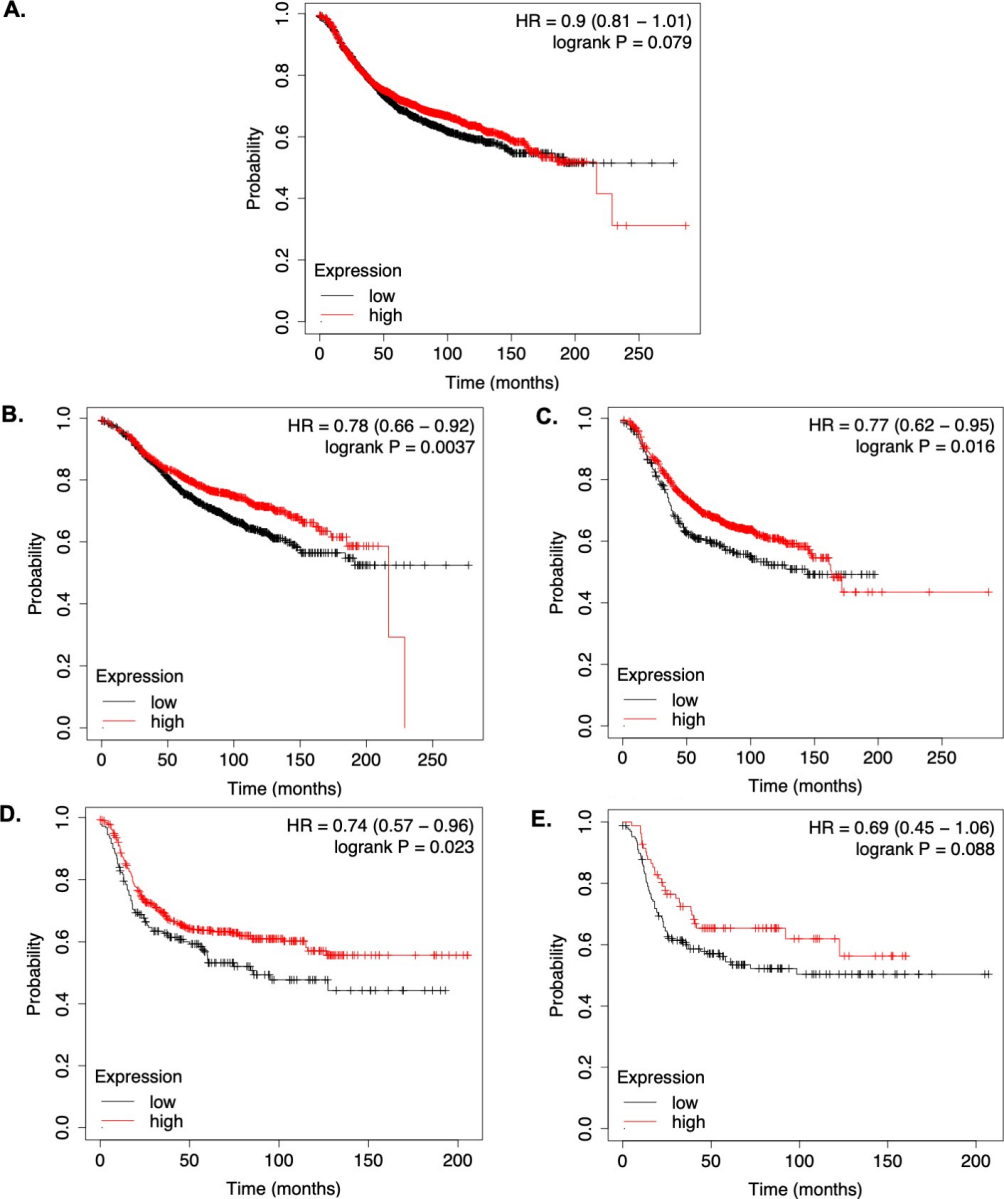
SK1 mRNA expression in breast cancer patients. Kaplan-Meier survival curves of *SK1* mRNA expression levels for (A) all breast cancer patients (n = 3,955), (B) luminal A (n = 1,933), (C) luminal B (n = 1,149), (D) HER2+ (n = 618) and (E) TNBC (n = 251) breast cancer subtypes. Auto select best cut-off was used to split the cohorts of patients by median expression values. p<0.05 was considered to indicate a statistically significant difference. HR, hazard ratio.

The increased metastasis in MMTV-PyMT SK1^-/-^ mice compared to SK1^+/+^ mice suggests that under specific conditions SK1 expression may be beneficial in the prevention of breast cancer progression. The prognostic values of *SK1* mRNA expression levels in patients with breast cancer were obtained from the Kaplan-Meier plotter website [31]. Analysis of *SK1* mRNA expression in all breast cancer patients did not show differences in relapse free survival (RFS) between patients with low and high *SK1* mRNA (Fig 5A). Remarkably, high *SK1* mRNA expression levels were observed to be significantly associated with better prognosis for patients with luminal A (Fig 5B) and luminal B (Fig 5C) and HER2+ (Fig 5D) breast cancer subtypes. Analysis of TNBC (Fig 5E) breast cancer subtypes did not show an association between *SK1* expression levels and RFS, but the number of patients was significantly lower compared to the other subtypes. These data indicate that high *SK1* mRNA expression may be associated with a good prognosis for breast cancer patients dependent on the subtype analyzed.

## Discussion

The results obtained in this study demonstrate that SK1 deficiency does not participate in tumor latency and growth in the MMTV-PyMT mouse breast cancer model, and unexpectedly increases lung metastasis in this model. The absence of differences in tumor growth between MMTV-PyMT SK1^-/-^ and MMTV-PyMT SK1^+/+^ mice was striking because of the large number of studies demonstrating the importance of SK1 in human breast cancer cell proliferation. Several reports have shown that SK1 promotes proliferation and survival of ERα-positive MCF-7 human breast cancer cells [16, 18, 19, 36]. However, the role of SK1 in proliferation of TNBC cell lines, where its expression and activity were shown to be higher [21, 37], is not completely clear yet. Early works have shown that SK1 is involved in TNBC MDA-MB-231 cell growth *in vitro* and in an *in vivo* xenograft SCID mouse model [37]. On the other hand, subsequent works have shown that SK1/S1P signaling is not critical for cell growth in MDA-MB-231 cells independent of EGF-mediated PI3K/AKT and ERK/MAP kinase pathways [20]. Additionally, a recent study has demonstrated that SK1 overexpression in MDA-MB-231 cells does not affect cell proliferation *in vitro* and, more importantly, orthotopically injecting these cells into mammary fat pads of nude mice generated tumors of similar size compared to control cells [21]. Similarly, there was no significant difference in primary tumor size or in tumor cell proliferation between control and SK1 knockdown in MDA-MB-231 cells. Gene-set enrichment analysis has shown that important pathways are upregulated in response to SK1 knockdown in prostate and breast cancer cells, like KRAS, IL2/STAT5, EMT and TNF/NFκB [38], indicating that switching off one pathway is insufficient to completely block cancer cell growth. Overall, these results suggest that the role of SK1 in regulating proliferation of breast cancer cells may be dependent on the molecular subtype and/or activation of independent pathways could overcome its loss.

As in human breast cancers, heterogeneity between tumors developed in MMTV-PyMT mice has been documented [25] with a significant proportion of MMTV-PyMT tumors having transcriptional profiles that are similar to HER2+ breast cancer as well as basal/TNBCs. The most recent data suggest that SK1 does not participate in cell growth of TNBC cells. Additionally, a complex interplay between HER2/SK1 has been described in MCF7 cells, where HER2 overexpression increases SK1 expression while SK1 downregulation induces expression of HER2 [13]. HER2 activation has an important role in breast cancer progression through the promotion of cell proliferation, possibly driving the increased frequency of larger tumor size observed in the HER2+ breast cancer subtype. SK1 absence in MMTV-PyMT tumors could increase HER2 expression, as was described previously in MCF7 cells, which offsets any effect of SK1 on proliferation. Further studies are necessary to determine if the predominance of HER2+ and basal/TNBCs in the MMTV-PyMT model could explain the absence of differences in tumor size between SK1^-/-^ and SK1^+/+^ condition.

Cancer metastasis is a complex multistep process involving local infiltration of tumor cells into the adjacent tissue, intravasation, survival in the circulatory system, extravasation and subsequent proliferation and colonization of competent organs [39]. In breast cancer cells, using stable isotope labeling by amino acids in cell culture (SILAC), SK1 has been shown to interact with key proteins that regulate cell motility (supervillin), cell adhesion and migration (myristoylated alanine-rich C-kinase substrate (MARCKS)-related protein) [40]. In MCF7 cells, SK1 downregulation significantly decreases EGF and prolactin-induced cell migration [18, 36]. Similarly, in a lung metastatic variant of the TNBC MDA-MB-231 cells, SK1 downregulation or its inhibition by PF-543 treatment, reduced EGF-induced cell migration [20]. In addition to its role in the intrinsic migration properties of breast cancer cells, SK1 expression has been shown to modulate angiogenesis and lymphangiogenesis, both important processes for cancer metastasis. *In vitro* models have demonstrated that S1P originated from breast tumor cells can initiate angiogenesis and lymphangiogenesis. SK1 downregulation in MDA-MB-231 cells decreased S1P secretion and reduced angiogenesis and lymphangiogenesis in a S1PR1-dependent manner and independent of VEGF factors [41]. Results generated from a syngeneic breast cancer model have corroborated that inhibition of SK1 in tumor cells decreased S1P levels and lung and lymph nodes metastasis through the disruption of angiogenesis and lymphangiogenesis [24]. The higher number of surface nodes and larger size of lung metastasis observed in MMTV-PyMT SK1^-/-^ compared to MMTV-PyMT SK1^+/+^ mice suggests that the effect of SK1 downregulation in breast cancer cell migration, and processes such as angio- and lymph-angiogenesis, are possibly exceeded by other processes regulating breast cancer metastasis in the MMTV-PyMT model analyzed.

Several studies have shown that metastasis requires close collaboration between cancer cells, inflammatory cells, and stromal elements, suggesting that cancer progression to metastasis may be associated with systematic inflammation [32]. Quantification of WBCs reflected a systematic inflammatory response associated with the metastasis progression in both, MMTV-PyMT SK1^-/-^ and MMTV-PyMT SK1^+/+^ mice. Although no statistical differences were noticed, the number of circulating WBCs trended higher for SK1^-/-^ (12330±6410) than for SK1^+/+^ (9350±5540) mice. An important parameter analyzed in breast cancer patients is the Neutrophil/Lymphocyte ratio (NLR). A higher NLR is present in patients with worse prognosis [33]. Higher NLR values were obtained for MMTV-PyMT SK1^-/-^ (1.5±1.03) compared to MMTV-PyMT SK1^+/+^ (1.18±0.94) mice though no statistical differences were observed. Even though SK1 deletion does not cause a significant change in the inflammatory response in terms of leucocytes number in the MMTV-PyMT model, this does not rule out the possible impact of SK1 deficiency on the levels of different cytokines (TNFα, TGFβ, IL6) that have been shown to participate in cancer metastasis [42, 43]. Additionally, further analysis is necessary to determine if SK1 deletion modified the immune response in the microenvironment of either the primary tumor or metastatic niche in the MMTV-PyMT model. The innate and adaptive immune system play a dynamic and critical role in breast cancer initiation and progression. In early pre-malignant lesions of MMTV-PyMT mice, macrophage infiltration promoted angiogenesis and metastatic potential [44]. Likewise, infiltration of CD4+ T lymphocytes enhanced malignant cell invasion and dissemination into peripheral blood and pulmonary metastasis [45]. The role of the immune cells is not limited to the primary site, but also implicated at the metastatic niche. In pre-metastatic lungs of MMTV-PyMT mice, neutrophil infiltration supported metastasis initiation and progression [46].

The increase in lung metastasis in MMTV-PyMT SK1^-/-^ mice may be driven by the lower S1P levels observed in these mice compared to the controls, and the known role of S1P in endothelial cell barrier function. S1P mediates cortical actin rearrangement and human endothelial cell barrier enhancement via S1PR1 signaling [35, 47]. Importantly, intravenous administration of S1P in mice reduces inflammatory histologic changes and microvascular leakage produced by LPS in lung parenchyma [48, 49]. These studies have demonstrated that S1P has a favorable effect on pulmonary endothelial cells through Rac activation and subsequent cytoskeletal remodeling [50]. Cancer cell metastasis is tightly linked to the maintenance of endothelium integrity [51, 52]. A decrease of S1PR1 signaling in lung endothelial cells of MMTV-PyMT SK1^-/-^ could result in an impaired pulmonary endothelial barrier and a consequent high number of lung nodes compared to the MMTV-PyMT SK1^+/+^ mice. Evaluation of Rac signaling and cytoskeletal proteins in the lung endothelial cells from MMTV-PyMT SK1^-/-^ mice would help to corroborate this hypothesis. In fact, lung colonization of MMTV-PyMT tumor cells was significantly enhanced after high dose W146 treatment (S1PR1 antagonist) in mice, suggesting that interruptions of the S1PR1-mediated vascular barrier functions can allow for metastasis in target organs by circulating tumor cells [53]. Additionally, platelets also play an important role in the maintenance of endothelial barrier [54] and thrombocytopenia significantly increases pulmonary vascular permeability reversed by restoring the circulating platelet population [55]. Blood platelet numbers in MMTV-PyMT SK1^-/-^ mice (582±80) showed values under the normal range (1275± 105) [56] although, not significant, they were lower compared to MMTV-PyMT SK1^+/+^ mice (798±95). This difference could also contribute to the increase lung metastasis observed in the MMTV-PyMT SK1^-/-^ mice.

Analysis of gene expression related with invasive and metastatic cancer in the primary tumor will also begin elucidating the mechanism by which SK1 absence increases lung metastasis in MMTV-PyMT mouse. Deletion of HIF1α in MMTV-PyMT mice caused a significant reduction in lung metastases, highlighting the role of this transcription factor in the progression of the disease [57]. In addition, deletion of MMP8 in the MMTV-PyMT model caused an early tumor onset and increase incidence of lung metastasis [58]. MMTV-PyMT crossed with cathepsin B knockout mice showed a delay in tumor onset and decreased metastasis compared to the WT mice [59]. Additionally, several studies have shown that PYMT tumor cells produce various immunosuppressive and chemiotactic molecules that modulate the tumor microenvironment and suppress the anti-cancer immune response to promote metastasis [60]. Ablation of STAT3 in the tumor epithelia of an inducible PyMT mouse model significantly increased immune cell infiltration in the primary tumor followed by tumor regression and absence of metastasis, suggesting that an immunosuppressive tumor microenvironment promoted by STAT3 is involved in modulating early tumor outgrowth and later metastasis [61]. Evaluation of the STAT3 pathway in the MMTV-PyMT SK1^-/-^ mice will be important, due that a positive feedback loop between SK/S1P/S1PR/STAT3 has previously been described in inflammation associated colon cancer [62–64] and a positive correlation between SK1 and STAT3 expression was stablished in ER-negative breast cancer [65].

Even though previous reports have shown a positive correlation between higher SK1 expression and worse prognosis in human breast cancer samples [12], in the present study the evaluation of survival in all breast cancer patients by Kaplan-Meier plot [31] did not indicate a connection between SK1 expression and RFS. In fact, high SK1 mRNA expression levels were associated with an improved prognosis in patients with luminal A, luminal B and HER2+ breast cancer subtypes, but no association was observed in TNBC. Differences between breast cancer subtypes were also described in other studies. SK1 expression was associated with shorter disease-free survival in ER+ patients treated with tamoxifen, but an inverse effect was detected within the subgroup of HER2+ ER+ samples [13, 14]. SK1 was also correlated with poor prognosis within the ER- HER2+ subtype; however, in ER- HER2- patients, the prognostic value of SK1 alone did not reach significance, being only associated with shorter disease-free survival if tumors also contained low levels of S1PR4 [66]. All these results indicate that the prognostic value of SK1 seems to be dependent on the human breast cancer subtype and in some cases this required the evaluation of additional genes (HER2 or S1PR).

In summary, in the MMTV-PyMT mouse breast cancer model, SK1 does not participate in breast tumor latency and growth, but its deletion significantly increases breast cancer metastasis to the lung, suggesting a metastasis-suppressing function for SK1 in this model of breast cancer. In fact, high *SK1* mRNA expression levels are associated with a better prognosis in patients with luminal A, luminal B and HER2+ breast cancer subtype, however the evaluation of all breast cancer patients did not indicate a connection between SK1 and RFS, suggesting that in certain subtypes of breast cancer patients *SK1* expression could be beneficial for the patients. The lower S1P blood levels observed in the MMTV-PyMT SK1^-/-^ mice compared to the controls could disturb the lung endothelial cell barrier function increasing the extravasation of breast cancer cells and the subsequent colonization of the lung. Further investigation into the role of SK1/S1P in the tumor microenvironment, a key player in both tumor initiation and breast cancer progression, as well as evaluation of Rac signaling in lung endothelial cells, from MMTV-PyMT SK1^-/-^ mice is important to understand the role of SK1 in spontaneous breast tumor initiation and breast cancer metastasis in the MMTV-PyMT mouse model.
